# Integrative Treatment of Reflux and Functional Dyspepsia in Children

**DOI:** 10.3390/children1020119

**Published:** 2014-08-18

**Authors:** Ann Ming Yeh, Brenda Golianu

**Affiliations:** 1 Department of Pediatrics, Stanford University, 750 Welch Road, Suite 116, Palo Alto, CA 94304, USA; 2 Department of Anesthesiology, Stanford University, 300 Pasteur Dr. Stanford, CA 94304, USA; E-Mail: bgolianu@stanford.edu

**Keywords:** reflux, dyspepsia, pediatric, integrative medicine, acupuncture, GERD

## Abstract

Gastroesophageal reflux disease (GERD) and functional dyspepsia (FD) are common problems in the pediatric population, with up to 7% of school-age children and up to 8% of adolescents suffering from epigastric pain, heartburn, and regurgitation. Reflux is defined as the passage of stomach contents into the esophagus, while GERD refers to reflux symptoms that are associated with symptoms or complications—such as pain, asthma, aspiration pneumonia, or chronic cough. FD, as defined by the Rome III classification, is a persistent upper abdominal pain or discomfort, not related to bowel movements, and without any organic cause, that is present for at least two months prior to diagnosis. Endoscopic examination is typically negative in FD, whereas patients with GERD may have evidence of esophagitis or gastritis either grossly or microscopically. Up to 70% of children with dyspepsia exhibit delayed gastric emptying. Treatment of GERD and FD requires an integrative approach that may include pharmacologic therapy, treating concurrent constipation, botanicals, mind body techniques, improving sleep hygiene, increasing physical activity, and traditional Chinese medicine and acupuncture.

## 1. Introduction

Gastroesophageal reflux is the normal physiologic passage of gastric contents into the esophagus. When reflux and regurgitation cause symptoms and complications, it is defined as gastroesophageal reflux disease, or GERD. Symptoms of GERD include heartburn, epigastric pain, feeding difficulties, dysphagia, and aerodigestive symptoms such as asthma, chronic cough, or recurrent pneumonia. On upper endoscopy, GERD patients exhibit erosive esophagitis or gastritis. Functional dyspepsia (FD) is defined by the ROME III criteria as a persistent upper abdominal pain or discomfort that: (1) is not exclusively relieved by defecation or associated with the onset of a change in stool frequency or stool form; and (2) organic disease is unlikely to explain the symptoms. The pain or discomfort in the upper abdomen has to be present at least once per week for at least two months prior to diagnosis. Endoscopic examination is negative in FD both grossly and microscopically. Dyspepsia is reported in 5%–10% of otherwise healthy adolescents [1].

Treatment for GERD symptoms often includes an empiric trial of acid suppression. Histamine-2 receptor antagonists and proton pump inhibitors are often effective in healing esophagitis and treating symptoms of reflux. However, acid suppressing medications have significant side effects or long-term risks that may limit their use. Pharmacologic therapies with acid suppression do not always effectively treat symptoms related to non-erosive reflux disease and reflux symptoms from non-acidic reflux. Therefore, treatment of GERD and FD suggests an integrative medicine approach.

Integrative medicine is a healing oriented medicine that takes account of the whole patient, including all elements of lifestyle and family health. It emphasizes the powerful triad of patient-family-practitioner, is informed by evidence, and makes use of all appropriate therapies. In patients with GERD and FD, an integrative medicine treatment plan may include botanicals, mind-body techniques, sleep hygiene, increasing physical activity, and acupuncture, in addition to pharmacologic therapies. This paper will illustrate the integrative medicine approach with a case study and review the current scientific evidence on integrative medicine treatments for GERD and FD in children and adolescents.

## 2. Case Report

An 11-year old girl presented to outpatient gastroenterology clinic with abdominal pain and chronic nausea for the past month. Her abdominal pain was daily, 3–4/10 in intensity, and in the epigastric area. She described the nausea as occurring almost every hour on a daily basis. She had one episode of non-bloody, non-bilious vomiting while at summer camp, but since then has had only chronic nausea. She reported several days of school absence in the last month. Her primary pediatrician initially started famotidine, which partially improved pain but not nausea. She had been constipated for several months, and strained often to stool. She denied diarrhea, fever, tenesmus, hematochezia, or pain on defecation. She reported no weight loss, normal PO intake and appetite. She recently restricted dairy, and although this change did not improve her pain, she described episodes where she felt particularly nauseated after eating ice cream. For breakfast, she usually ate toast pops or cereal with fruit. Lunch was the typical school hot lunch with could include pizza, spaghetti, chicken nuggets, or a sandwich. Dinner was often eaten at home, and parents reported a healthy home-cooked organic diet that included meat.

Her past medical history was negative for surgeries, hospitalizations, or major infections. Her mother also has a history of constipation. She was in the 6th grade and with good grades, and had a sister in college. Parents both worked for a successful company they started together and endorsed increased work stress at home. She slept well from 9:30 pm to 6:30 am each night, was rested upon waking, but sometimes had trouble falling asleep. She described herself as a worrier and the caretaker of the family. Both mom and patient recognized that she could be sensitive, and she felt sad and worried when her parents were away. She described herself as easygoing with other kids in her class. Her main physical activity was during physical education class at school, and she did not exercise much otherwise. 

On physical exam, her height is the 25th percentile, weight was at the 8th percentile, and BMI was at the 10th percentile. Abdominal exam was notable for hard stool palpable in the right ascending colon, and mild epigastric tenderness. Her physical exam was otherwise benign. Infectious stool studies for salmonella, shigella, campylobacter, Escherichia coli, giardia, cryptosporidium, and ova and parasites were all negative.

Her initial treatment plan included a trial of acid suppression with lansoprazole 30 mg a day for 4 weeks. She was also started on Miralax at 8.5 g–17 g/day to titrate to soft mushy stools and to increase fiber and fluids in her diet. She was advised to continue diary restriction and add a calcium supplement of 1000 mg a day. In addition, a trial of 0.5 mg of melatonin at bedtime was suggested to help with sleep latency. She was instructed to do moderate physical activity daily outside of PE class. Her nausea and epigastric pain did not improve with these initial measures, and she underwent upper endoscopy with biopsies. Endoscopy showed normal anatomy, mucosa and pathology and patient was subsequently given the diagnosis of functional dyspepsia.

Lansoprazole was discontinued given her lack of improvement, and parents were introduced to integrative treatment modalities. She started acupuncture treatments every other week along with a trial of gut-directed clinical hypnosis.

On traditional Chinese medicine (TCM) exam, her tongue exam revealed a tongue with a thin white coating that was dry appearing, scalloping at the lateral tongue edges and a puffy red and pink tip. Her pulse exam revealed a soft deep pulse at the third positions bilaterally, an empty pulse at the second position on the right, and wiry pulse at the second position on the left. This indicates potential TCM diagnosis of SP and KI Qi deficiency. Her acupuncture treatments consisted of Seirin J-type (0.16 × 30 mm) needles inserted at ST36 and LI4 points bilaterally. Alcohol prep was used on the skin prior to insertion of needles. Pointer Plus ear point finder was used to find reactive auricular acupuncture points and gold beads were placed at the ear points “esophagus”, “spleen”, and “liver” on one ear, alternating sides at the following visit. Body magnets were placed at SP9, SP6, PC6, TH5, and ST43 bilaterally. Patient was instructed to remove magnets and beads in 3 days unless they fell off sooner. In addition, she was instructed to apply additional magnets on points PC6 and ST43 on an as needed basis at home. These acupuncture and acupressure points were chosen to treat constipation, to clear inflammation, and to tonify SP and KI Qi. Points PC6 and ST43 in particular were chosen to treat nausea. At our institution, nausea and dyspepsia are commonly treated with the points listed above; a standard combination includes PC6 and ST43 (nausea), LI 4 and ST 36 (if constipation plays a role), SP6 and SP9 (SP qi deficiency), and CV 10 and 12 (dyspepsia). The choice of acupuncture *versus* acupressure and exact combination of points depends on patient tolerance and the patient’s overall constellation of symptoms. This patient specifically did not desire any abdominal points, and therefore CV10 and CV12 were avoided.

Her gut-directed clinical hypnosis sessions were particularly helpful, and she was able to record the sessions on her tablet computer and use them as needed at home. During her hypnosis sessions with her trained therapist, she revealed that family discord and stress from her parents significantly worsened her symptoms. Parents received counseling from the therapist and tried to avoid stressful conversations in front of the patient. She was also started on ginger chews, ginger tea, and deglycyrrhizinated licorice on an as needed basis for nausea. Physical activity was encouraged, and she reported that walking after meals helped relieve postprandial fullness.

After a course of acupuncture, hypnotherapy, and the above interventions, she felt significant improvement in symptoms, with minimal nausea, excellent weight gain, and no further school absences. When she did feel more nauseated, she would self-apply acupressure magnets or listen to pre-recorded clinical hypnosis sessions as needed.

## 3. Treatment of GERD and Dyspepsia

### 3.1. Pharmacologic

Medications for GERD include histamine-2 receptor antagonists, proton pump inhibitors, and mucosal surface barriers and gastric acid buffering agents. Prokinetics are less frequently used.

First line medications include histamine-2 receptor antagonists (H2RA’s) such as ranitidine, cimetidine, famotidine, and nitazidine. The histamine-2 receptors are found on the acid producing parietal cells of the stomach mucosa, and blockage of these receptors partially decreases production of stomach acid, typically within 30 min of administration, with the effect lasting for 6 h [2]. Tachyphylaxis, or a tolerance to the medication and a decreased treatment response, occurs after several weeks and thereby may limit long-term use [3].

Proton pump inhibitors (PPI’s) such as omeprazole, esomeprazole, and lansoprazole are second line agents for increased acid suppression. These medications block acid secretion by inhibition of the sodium-potassium-ATPase pump. PPI’s are superior to H2RA’s in acid suppression and for erosive esophagitis. The efficacy of PPI’s also does not decrease with long-term use, as compared to H2RA’s. In older children and adolescents, a patient with classic GERD symptoms may warrant an empiric trial of acid suppression to determine if symptoms are related to acid reflux. In an older child or adolescent with symptoms suggestive of GERD, The North American Society of Pediatric Gastroenterology and Nutrition (NASPGAN) clinical guidelines recommend an empiric 4 week trial of a proton pump inhibitor (PPI) to determine if symptoms respond [4]. However, treatment response does not confirm a GERD diagnosis because it may reflect a spontaneous resolution of symptoms or a placebo. Upper endoscopy may be recommended to confirm diagnosis if patient does not respond to empiric acid blockade or if unable to wean off medication.

The most common reported side effects of acid suppressing medications include constipation, headache, nausea, and diarrhea, which occur with an incidence of 2%–7% [4]. Gastric acid serves as a part of the body’s innate immune system. Reviews of the pediatric literature also raise concerns for an increased incidence of infections in patients exposed to acid suppression [5]. Previously healthy pediatric patients diagnosed with GERD and treated with either ranitidine or omeprazole had a higher incidence of pneumonia (12%* vs.* 2%, *p* < 0.05) and acute gastroenteritis (42% *vs.* 20% *p* = 0.001) during the 4 month follow up compared to healthy controls [6]. Adult studies also associate long-term PPI use with increased risk of nutrient malabsorption for calcium, iron, magnesium and vitamin B12. Because of decreased calcium or B12 absorption, an increased fall and fracture risk is also reported in the elderly population after long-term (>1 year) PPI use [7,8]. Furthermore, after PPI treatment in healthy volunteers for 8 weeks, PPI’s induced acid related withdrawal symptoms secondary to rebound acid hypersecretion [9]. Patients often report difficulty weaning off PPI’s secondary to rebound symptoms.

There is little evidence to justify the treatment of GERD and dyspepsia with promotility agents such as cisapride, erythromycin and metoclopramide. Although effective at increasing gastric motility and decreasing reflux symptoms, cisapride, a serotonin receptor agonist, was withdrawn from the market in the United States due to complications with fatal arrhythmias, long QT syndrome, and sudden death. Erythromycin, an antibiotic, also has promotility effects when administered at low doses (1–3 mg/kg) to stimulate gastric antral motility. However, the studies are limited to patients with delayed gastric emptying and dysmotility, and to date there is insufficient evidence for treating GERD and dyspepsia [4,10]. Metoclopromide is also a dopamine antagonist that has been shown to decrease reflux index and daily symptoms [11]. However, given the potential for extrapyramidal side effects, including irreversible tardive dyskinesia, the side effect profile of metoclopromide greatly limits its use in clinical practice [12,13].

Antacids buffer gastric acid contents, and decrease reflux symptoms and can aid in healing erosive esophagitis. However, formal studies using pH or impedance monitoring have not been performed in children. Mucosal surface protective agents with alginate or sucralfate also have limited evidence in pediatrics, though sucralfate may be as effective as cimetidine for healing erosive esophagitis. Long-term sucralfate use should be used with caution due to unknown risk of aluminum toxicity in children.

### 3.2. Diet

Patients with reflux often benefit from a diet that avoids specific food triggers. In particular, fatty foods, spicy foods, acidic foods, chocolate, and caffeine worsen reflux symptoms. Some patients with food sensitivities or an allergic component to their symptoms may benefit from an elimination diet. Elimination diets systematically remove and then reintroduce common allergic triggers such as dairy, wheat, egg, nuts, or fish to look for symptom improvement upon elimination and symptom worsening upon reintroduction. Prior to completely eliminating wheat, a celiac screen should be performed with a tissue transglutaminase IgA antibody, and a total IgA level.

### 3.3. Other Motility Concerns: Constipation

Constipation and fecal retention can worsen GERD and dyspepsia symptoms in all age groups, and constipation and reflux often co-exist. In a study of children aged 4–16 years old with functional constipation diagnosed by Rome III criteria, approximately 40% of these patients presented with pathologic acid reflux by 24 h esophageal pH monitoring [14]. Constipation can also significantly affect overall GI motility. Rectal distension impaired gastric slow waves in healthy volunteers [15]. An association with reflux symptoms and constipation may be particularly prominent in children with underlying neurologic problems, underlying motility problems, or in patients with dysmotility after infectious enteritis [16]. Therefore, treating concurrent constipation with diet or laxatives can improve reflux symptoms.

### 3.4. Botanicals and Supplements

Various botanicals have been studied in the treatment of GERD and functional dyspepsia. Although studies on herbal remedies are small and with varied results, certain botanicals such as Iberogast (STW-5), deglycyrrhizinated licorice, and ginger show good potential for treating reflux symptoms with few adverse effects. Patients with severe reflux should be cautioned against using non-enteric coated peppermint, as it has been shown to relax the lower esophageal sphincter and potentially worsen reflux symptoms [17].

#### 3.4.1. Iberogast

Iberogast (STW-5—Medical Futures Inc., Richmond Hill, Ontario, Canada) is a commercial preparation of 9 herbal extracts including bitter candy tuft, lemon balm leaf, chamomile flower, caraway fruit, licorice root, angelica root, milk thistle fruit, peppermint leaf, and greater celandine herb. In vitro, it has been shown to protect against the development of ulcers with decreased acid production, increased mucin production, an increase in prostaglandin E2 release, and a decrease in leukotrienes [18]. Although the evidence is mixed, clinical studies suggest that it acts directly to increase gastric motility in healthy subjects by increasing the motility index of antral pressure waves [19], but not necessarily decrease the overall gastric emptying time [20]. A review of the 12 clinical studies on Iberogast concluded that it is both safe and effective for treatment of functional dyspepsia and irritable bowel syndrome. The incidence of adverse drug reactions in this review was reported to be 0.04% and was mainly in the form of hypersensitivity reactions such as skin irritation, dyspnea, and pruritis [21]. The low incidence of adverse events and excellent safety profile is confirmed by the spontaneous reporting system in Germany and worldwide since the product was introduced approximately 50 years ago. Although no studies on efficacy exist in children, the preliminary adult studies and excellent safety profile are encouraging.

#### 3.4.2. Licorice

Licorice root, the dried rhizome or extracts of *glycyrrhiza glabra*, has long been used in botanical medicine for treatment of gastric inflammation. The mechanism of action is thought to be due to inhibition of prostaglandin synthesis and lipoxygenase [22]. Glycyrrhizin has mineralocorticoid properties, and therefore the deglycyrrhizinated form of licorice is recommended for long-term or higher doses. Deglycyrrhizinated licorice (DGL) and licorice extracts without the glycyrrhizin do not have side effects of hyperkalemia, hypertension, and sodium retention [22]. In a small, randomized, double-blind, placebo controlled study of 50 adults with functional dyspepsia as diagnosed by Rome III criteria, subjects were randomized to placebo or a 75 mg extract of *Glycyrrhiza glabra* (GutGard^®^, Karnataka, India) for 30 days. Symptoms were assessed with a 7-point Likert scale of dyspepsia symptom severity at day 0, 15, and 30. Compared to placebo, the licorice extract showed a significant decrease in total symptom scores (*p* < 0.05) and improvement in quality of life [23]. Although more evidence is needed, integrative medicine practitioners frequently use deglycyrrhizinated licorice to help wean off acid suppression.

#### 3.4.3. Ginger

Ginger root, the rhizome of *Zingiber officinale*, has been used traditionally as a kitchen spice but also for treating reflux symptoms and dyspepsia. Adult studies have demonstrated efficacy of ginger to treat pregnancy-induced nausea and vomiting, postoperative nausea, and drug induced nausea and vomiting [24,25,26,27,28]. Ginger root has a prokinetic effect that may be mediated by cholinergic action and spasmogenic properties that have been demonstrated in mouse and guinea pig models [29,30]. In healthy volunteers, both Wu [31] and Micklefield [32] showed ginger root improved gastric emptying and gastroduodenal motility in both the fasting and fed state. The recommended dose ranges from 1 g to 1.5 g of the dried herb per day, with administration typically 30 min to 1 h before a meal. It is important to note the ginger rhizome extract is much more concentrated than the dried ginger root powder. When used in typical doses, most patients tolerate ginger well. Side effects are reported when doses exceed 5 g/day and include heartburn, abdominal discomfort and diarrhea. Ginger root has been shown to have an antiplatelet effect due to its ability to inhibit platelet thromboxane [33]. Therefore, it is relatively contraindicated in patients with a bleeding disorder, and should be discontinued prior to surgical procedures.

In terms of safety in pediatrics, no trials to date have investigated ginger and safety in children; though the use of ginger in pregnant women is likely safe with no increased risk of congenital malformations or harm to the fetus. In children who are unable to swallow ginger root capsules, ginger candies, chews and teas are often more palatable.

#### 3.4.4. Peppermint

Although there is some evidence that enteric coated peppermint oil may be effective in treating irritable bowel syndrome in adults and in children [34,35], caution should be used for peppermint oil in patients with reflux. Peppermint is known to have anti-spasmodic activity on smooth muscles, and may increase relaxation of the lower esophageal sphincter. This could exacerbate reflux symptoms, especially in patients with erosive esophagitis or hiatal hernia [17].

### 3.5. Sleep Hygiene and Melatonin

Melatonin is a naturally occurring hormone produced by the pineal gland in the brain. It is produced with the onset of darkness and inhibited by exposure to light on the retina. It regulates the sleep-wake cycle and serves as a darkness signal but is not sedating. Melatonin levels are low in the daytime. Endogenous production rises at night and peaks between 11 pm and 3 am [36].

In addition to its effects on the sleep-wake cycle, emerging evidence suggests that melatonin also has a gastroprotective effect. Melatonin production by the enterochromaffin cells in the digestive mucosa exceeds pineal gland production after tryptophan stimulation [37,38]. Melatonin in the GI tract has been shown* in vitro* to regulate GI motility, modulate visceral sensation, and produce an anti-inflammatory response [39].

A study by Klupinska* et al.* [40] found that patients with GERD and recurrent peptic ulcer disease had decreased levels of nighttime peak melatonin compared to those with non-erosive reflux disease and functional dyspepsia, suggesting a protective effect of melatonin on the upper GI mucosa. A small randomized controlled trial of 27 adults with GERD found that 3 mg of melatonin alone at bedtime was effective in treating GERD symptoms over placebo [41]. Another small trial by Kandil *et al*. [41] randomized GERD patients into 4 treatment groups: placebo, 3 mg of immediate release melatonin, 20 mg of omeprazole, or melatonin plus omeprazole for 8 weeks. Although omeprazole alone was more effective at symptom improvement than melatonin alone, melatonin alone was more effective than placebo. Furthermore, the addition of melatonin to omeprazole provided a synergistic effect and increased the efficacy of omeprazole. Interestingly, patients in both melatonin groups had significantly higher LES sphincter pressures on manometry compared to both the omeprazole only group and controls. Another head-to-head trial compared 20 mg of omeprazole to a supplement containing melatonin (6 mg), its precursor L-tryptophan (200 mg), B vitamins and folic acid for 40 days in 350 patients with GERD. 100% of patients in the supplement group had regression of symptoms, compared to 66% of the omeprazole group [42]. All studies reported no significant side effects or complications of melatonin supplementation. Although these adult studies are small and need further replication, they suggest a significant gastroprotective effect of both endogenous and exogenous melatonin.

In children with delayed sleep phase disorder and prolonged sleep latency, short-term supplementation of melatonin at low doses in children is generally regarded as safe and well tolerated [43,44,45]. Side effects include early morning grogginess, somnolence, dizziness and headaches. Effective dosing in children ranges from 0.3 mg to 5 mg, and oftentimes doses of 0.3 mg to 1 mg are sufficient to improve sleep latency. Further research is needed to investigate the role of melatonin and efficacy on the GI tract diseases in children.

Sleep disruptions can significantly decrease normal melatonin production and thereby affect the gastroprotective effect on the mucosal lining. Poor sleep quality is associated with increased acid exposure, increased exacerbations of reflux the following day, and visceral hyperalgesia [46,47]. Working to improving the child’s sleep hygiene may help increase the physiologic production of melatonin and decrease visceral hypersensitivity. Important elements of a sleep history include sleep quality, sleep latency (time it takes to transition from full wakefulness to sleep), night waking, daytime drowsiness, and exposure to light at night. Improving a patient’s sleep hygiene includes eliminating use of electronics one hour prior to bedtime, earlier bedtime, and improving the overall quality of sleep.

### 3.6. Acupuncture and Acupressure

Acupuncture, a healing modality used in traditional Chinese medicine, uses fine needles inserted at defined acupuncture points to balance the body’s Qi, or life energy. In traditional Chinese medicine, a patient is evaluated for imbalances or blockages in the body’s Qi, and acupuncture is employed as part of a holistic treatment approach. Treatments may include Chinese herbs, massage, and movement-based therapies such as Tai Qi or QiGong. Specifically for reflux and dyspepsia, certain acupuncture points such as PC6 (*neiguan*), and ST36 (*zusanli*) have been effective in improving reflux symptoms, nausea, and vomiting. Research on these two points has also started to pinpoint the physiologic mechanism of action.

PC6 (*neiguan*), on the pericardium meridian, is one of the most used and investigated acupuncture points for nausea, vomiting, and reflux. The point is located in the groove caudal to the flexor carpi radialis and cranial to the superficial digital flexor muscles. Acupuncture and electrical stimulation of PC6 has been shown to be as effective as antiemetics in adults with nausea and vomiting induced by chemotherapy, pregnancy, and postoperative settings [48,49,50]. In children, both auricular acupuncture and body acupuncture have improved postoperative and chemotherapy induced nausea and vomiting [51,52,53,54]. Furthermore, functional MRI studies have shown increased attenuation of the cerebrocerebellum after acupuncture on point PC6 over control points, suggesting modulation of cerebellar activities [55].

ST36 (*zusanli*), on the stomach meridian, is located at the proximal one-fifth of the craniolateral surface of the rear leg, distal to the head of the tibia in a depression between the muscles of the cranial tibia and the extensor digitalis longus. In healthy men, electrical acupuncture at ST36 decreases basal acid output and gastric acid secretion, increases pancreatic polypeptide levels, and increases amplitude of gastric antral contractions [56].

Other acupuncture points that have been traditionally used for GERD and dyspepsia include the following points, especially if found to be tender to touch: ST43 (*xiangu*), CV12 (*zhongwan*), ST25 (*tianshu*), SP4 (*gongsun*), LV3 (*taicong*), BL21 (*weishu*), and LI4 (*hegu*). The above points may also be used in combinations in a clinical treatment, as indicated by the presenting complaint and Chinese medical pattern diagnosis. However, these individual points have not been studied extensively in clinical studies, and further research is required to evaluate efficacy.

There are no published clinical trials to date on acupuncture and acupressure in children with reflux or dyspepsia. However, the safety of acupuncture in children is well documented. One review of the literature cites a 1.55 risk of any adverse events occurring in 100 treatments of acupuncture [57]. Puncture redness is the most commonly reported side effect, followed by needle pain, and light-headedness. A serious adverse event is defined as an event that is life threatening or requires hospitalization. Studies have reported this risk to be as low as 0.05/10,000 treatments in the general population [57]. In addition to acupuncture, non-invasive forms of acupressure are available for pediatrics using laser acupuncture, topical magnets, and acupressure beads. These may be preferred by needle phobic children or in high-risk patients who may be immunosuppressed or at a risk for bleeding. They may also be used as adjunctive treatments following needle placement.

### 3.7. Mind Body Therapy

Psychosocial stressors may exacerbate symptoms in many children with GERD and FD. Compared with healthy controls, FD patients are more likely to exhibit psychological distress, somatization, anxiety and depression [58]. Adult patients with dyspepsia also have a higher reported incidence of childhood emotional abuse [59]. It is therefore important to address the mind-body-gut connection when treating a patient with GERD or FD. Patients with significant anxiety and depression may benefit from psychiatric evaluation or counseling. All children with increased psychosocial stressors and mild anxiety can benefit from mind-body therapies and relaxation techniques. Types of mind-body therapy include mindfulness meditation, guided imagery, biofeedback, clinical hypnosis, and yoga. Treatment can be tailored to the interest and motivation of the individual patient.

Gut directed hypnotherapy is a form of clinical hypnosis that is based on muscular and mental relaxation. General hypnotic suggestions are used to either focus on the symptoms or to distract from them. A Cochrane review in 2007 found hypnotherapy to be effective for irritable bowel syndrome in adults [60]. Hypnotherapy was effective in providing long-term symptom improvement and decreased medication use and consultation [61]. A small study of gut-directed hypnotherapy was found to shorten gastric emptying both in dyspeptic and in healthy subjects as measured by ultrasonography [62]. In children with functional abdominal pain or irritable bowel syndrome, three randomized control trials have been published to date. Van Tilburg* et al.* [63] established that a home-based audio of guided imagery recordings using hypnotherapy techniques was more effective than standard care to improve pain symptoms in children with functional abdominal pain (63% *vs*. 27%). Studies by Weydert [64] and Vlieger [65] also demonstrated that gut directed hypnosis by a trained therapist was superior to standard care in pediatric patients with irritable bowel syndrome. Although all three of the above studies had small sample sizes (ranging from 22 to 52 patients), the efficacy results and excellent safety profile suggest that it is a likely beneficial treatment modality [66].

### 3.8. Obesity and Weight Loss

Obesity may predispose a patient to increased intragastric pressure, more frequent transient lower esophageal sphincter relaxations, and increased esophageal acid exposure. Although data is scarce in the pediatric population, adult studies have shown obesity to be correlated with increased incidence of GERD, Barrett’s esophagus, and esophageal adenocarcinoma [67,68]. Jacobson’s study of women with GERD symptoms showed that even moderate weight gain in person of normal weight caused or exacerbated reflux symptoms. A large prospective population-based cohort study further indicates that weight loss in patients with GER symptoms was dose-dependently associated with a symptom reduction. Weight loss was also associated with increased treatment success with antireflux medication [69]. Recommending weight loss or weight maintenance in obese and overweight children, and encouraging moderate physical activity in all children with GERD and FD should be an integral component to the treatment plan.

## 4. Conclusions

As illustrated by the case report and review of literature, patients with GERD and FD can benefit significantly from an integrative treatment approach. Pharmacologic medications such as H2-blockers and PPI's are effective at treating symptoms. However, side effects, long-term risks, and difficulty with discontinuation can limit their use. Botanicals such as Iberogast, DGL, and ginger can often provide adjunct symptomatic relief with a good safety profile. Emerging evidence suggests good sleep hygiene and melatonin secretion may play an important gastroprotective role in the GI tract. Furthermore, working with mind-body therapies and acupuncture may improve GERD symptoms, motility, and help with stress reduction. We encourage all pediatricians and pediatric gastroenterologists to consider an integrative and holistic approach when treating a patient with gastroesophageal reflux and functional dyspepsia.
